# Low Intensity Respiratory Muscle Training in COVID-19 Patients after Invasive Mechanical Ventilation: A Retrospective Case-Series Study

**DOI:** 10.3390/biomedicines10112807

**Published:** 2022-11-04

**Authors:** Koldo Villelabeitia-Jaureguizar, César Calvo-Lobo, David Rodríguez-Sanz, Davinia Vicente-Campos, José Adrián Castro-Portal, Marta López-Cañadas, Ricardo Becerro-de-Bengoa-Vallejo, José López Chicharro

**Affiliations:** 1 Infanta Elena University Hospital, Valdemoro, 28342 Madrid, Spain; 2 Faculty of Nursing, Physiotherapy and Podiatry, Universidad Complutense de Madrid, 28040 Madrid, Spain; 3 Faculty of Health Sciences, Universidad Francisco de Vitoria, Pozuelo de Alarcón, 28223 Madrid, Spain

**Keywords:** COVID-19, respiratory muscle training, invasive mechanical ventilation, dyspnea

## Abstract

Worldwide, healthcare systems had to respond to an exponential increase in COVID-19 patients with a noteworthy increment in intensive care units (ICU) admissions and invasive mechanical ventilation (IMV). The aim was to determine low intensity respiratory muscle training (RMT) effects in COVID-19 patients upon medical discharge and after an ICU stay with IMV. A retrospective case-series study was performed. Forty COVID-19 patients were enrolled and divided into twenty participants who received IMV during ICU stay (IMV group) and 20 participants who did not receive IMV nor an ICU stay (non-IMV group). Maximal expiratory pressure (PE_max_), maximal inspiratory pressure (PI_max_), COPD assessment test (CAT) and Medical Research Council (MRC) dyspnea scale were collected at baseline and after 12 weeks of low intensity RMT. A greater MRC dyspnea score and lower PI_max_ were shown at baseline in the IMV group versus the non-IMV group (*p* < 0.01). RMT effects on the total sample improved all outcome measurements (*p* < 0.05; *d* = 0.38–0.98). Intragroup comparisons after RMT improved PI_max_, CAT and MRC scores in the IMV group (*p* = 0.001; *d* = 0.94–1.09), but not for PI_max_ in the non-IMV group (*p* > 0.05). Between-groups comparison after RMT only showed MRC dyspnea improvements (*p* = 0.020; *d* = 0.74) in the IMV group versus non-IMV group. Furthermore, PI_max_ decrease was only predicted by the IMV presence (*R*^2^ = 0.378). Low intensity RMT may improve respiratory muscle strength, health related quality of life and dyspnea in COVID-19 patients. Especially, low intensity RMT could improve dyspnea level and maybe PI_max_ in COVID-19 patients who received IMV in ICU.

## 1. Introduction

In the last decades, an increased survival of critical patients was achieved due to the rising use of invasive mechanical ventilation (IMV). Nevertheless, a general muscle affectation of critical patients was presented as a consequence of the greater use of IMV, named deconditioning syndrome, which was typically characterized by skeletal and respiratory muscle weakness [1,2]. To date, diaphragm dysfunction as a progressive reduction in muscle strength was induced immediately after IMV application, being a relevant clinical problem in intensive care units (ICUs) [3,4,5], as well as worsening the prognosis and mortality of pathological patients in ICUs [6,7,8,9,10].

Diaphragm atrophy induced by IMV occurred early [11] and may be detectable in patients at 24–72 h after IMV application [12], being atrophy of a greater extent than associated atrophy within skeletal musculature disuse [13]. Especially, atrophy affected type-II muscle fibers in the beginning of the disease process [14,15]. Histologically, remodeling processes with increased hybrid fibers in conjunction with type-I fiber detriment may be identified at later stages [16]. Indeed, ultrastructural alterations such as myofibrillar disorganization, Z lines alteration and increased cytoplasmic lipid vacuoles [17,18], as well as contractile dysfunction, were previously reported [11]. These changes did not only affect the diaphragm but the intercostal muscles were also affected to a lesser extent [15,19].

Nevertheless, it is worth noting that atrophy may not explain strength loss in an isolated manner; due to strength being normalized by isolated muscle fiber cross sectional areas, namely, tension, this weakness was disproportionately greater than the weakness justified only by muscle atrophy [11,18,20].

Diaphragm atrophy and dysfunction secondary to IMV primarily occurred due to decreased protein synthesis, increased proteolysis and mitochondrial functional alterations that caused an excess production of reactive oxygen species (ROS), increasing oxidative stress at the diaphragmatic level [12,21]. Indeed, recent data reported a wide reduction in diaphragm blood flow in patients who suffered from deconditioning syndrome [22], causing speculation that the reduction in oxygen availability may cause the formation of ROS within consequent proteolysis and oxidative stress [23].

Patients who suffered from diaphragmatic dysfunction linked to IMV displayed decreased values of trans-diaphragmatic pressure (Pdi) [24], and this reduction was logarithmically increased by days spent on IMV [25]. Indeed, maximal inspiratory pressures (PI_max_) were decreased in parallel with the trans-diaphragmatic pressure.

Physical therapy rehabilitation strategies in ICUs were mainly focused on prevention or reduction of peripheral muscle dysfunction [26,27,28,29], while respiratory muscles received little attention [30]. Thus, inspiratory muscle training (IMT) was particularly focused on diaphragm and accessory inspiratory muscles in order to improve muscle strength and resistance [30]. A systematic review that examined research carried out with IMT in critical patients ventilated mechanically identified a lack of clinical trials about this topic [31]. Authors from this systematic review estimated that IMT significantly increased the strength of inspiratory muscles.

As a result of RMT, the improvement of inspiratory muscles strength was also linked to a quality of life improvement at 2 weeks after training in patients whose mechanic ventilation was retired [32]. Due to the reversibility of inspiratory muscle weakness and the potential benefits of RMT on patients’ quality of life, clinical staff from ICUs intended to transfer this scientific evidence to clinical practice [33].

From February to March 2020, the novel SARS-Cov-2 coronavirus spread and became a worldwide pandemic. Healthcare systems from all countries had to respond to an exponential increase in patients affected by COVID-19 in a short time, in conjunction with a noteworthy increment in ICU admissions and, frequently, IMV.

Therefore, the aim of this research was to determine the RMT effects in COVID-19 patients, established upon medical discharge and after a period of ICU stay with IMV.

## 2. Methods

### 2.1. Study Design

A case-series study in COVID-19 patients was retrospectively carried out in the Rehabilitation Service of the Infanta Elena University Hospital (Valdemoro, Spain) from March to November 2020, according to the CARE Guidelines for the Consensus-based Clinical Case Reporting Guideline Development [34].

### 2.2. Ethical Requirements

This research was approved by the Jiménez Díaz Foundation Ethic Committee (Approval Code: CEIm-FJD 02/21; Madrid, Spain). In addition, participants’ data were retrospectively obtained from the medical records of the Rehabilitation Service of the Infanta Elena University Hospital (Valdemoro, Spain), and an informed consent form was not necessary to participate in this research according to the protocol approved by the ethic committee. All ethical requirements such as the Helsinki Declaration and Human Rights for biomedical investigation were respected [35,36].

### 2.3. Participants

Inclusion criteria comprised patients with post-COVID-19 confirmed infection from the rehabilitation Service of the Valdemoro University Hospital (Valdemoro, Spain) who had received RMT during 12 weeks from March to November 2020. Patients’ data were obtained from medical records in order to check the compliance with inclusion and exclusion criteria. In addition, inclusion criteria for the IMV group required participants who had recovered from the COVID-19 condition and who stayed in the ICU for at least 6 days, as well as received IMV, while the non-IMV-group comprised participants who had recovered from the COVID-19 condition without an ICU stay and did not receive IMV. Exclusion criteria comprised patients with comorbid medical conditions (i.e., neurological diseases) or participants who were under some sedative or paralytic agents.

Forty COVID-19 patients who met the inclusion criteria were enrolled in the study and divided into twenty participants who received IMV during an ICU stay and twenty participants who did not receive IMV nor an ICU stay. Descriptive data and outcome measurements were obtained at baseline and outcome measurements were also registered after 12 weeks of RMT. Baseline characteristics of the 40 COVID-19 patients are outlined in Table 1 and Table 2.

### 2.4. Descriptive Data

Quantitative descriptive data comprised age (years), hospitalization (days), ICU stay (days), diffusing capacity for carbon monoxide (DLCO; %), diffusing capacity divided by the alveolar volume (DL/VA; %), forced vital capacity (FVC; %), forced expiration volume in the 1st second (FEV1; %) and Tiffeneau index (IT; %). DLCO refers to the amount of carbon monoxide that passes through the alveolar capillary membrane into the capillary blood per unit time and per unit pressure difference with a percentage of the measured value to the predicted value >80% as normal [37]. FVC, FEV1 and IT reflected lung function and showed good correlation with chest wall expansion (0.747) and reliability (0.786–0.929) [38,39]. All procedures were executed according to the American Thoracic Society (ATS) and European Respiratory Society (ERS) guidelines [40,41].

Categorical descriptive data comprised sex (male/female) and presence (yes/no) of hypertension, diabetes, dyslipidemia, coronary artery disease (CAD), chronic obstructive pulmonary disease (COPD), smoking, obesity, chronic kidney disease (CKD) and hypothyroidism according to the medical record.

### 2.5. Outcome Measurements

Maximal expiratory pressure (PE_max_), measured in cmH_2_O and %; maximal inspiratory pressure (PI_max_), also measured in cmH_2_O and %; health related quality of life, measured by the COPD assessment test (CAT) score; and dyspnea level, assessed by the Medical Research Council (MRC) scale score, were collected at baseline and after 12 weeks of RMT for all COVID-19 patients. PI_max_ measured in % was considered the main outcome measurement and the rest of the variables as secondary outcome measurements.

#### 2.5.1. Respiratory Muscle Strength

Respiratory muscle strength was assessed by measuring PI_max_ and PE_max_ using a POWERbreathe^®^ KH1 device (Powerbreathe International Ltd., Southam, UK) from residual volume and total lung capacity, respectively, according to the rules of the American Thoracic Society and European Respiratory Society [40,41]. Each measurement was obtained in the reference unit of centimeter of water column (cm H_2_O). The procedure was repeated at least three times or until two reproducible efforts were recorded (i.e., within 5% of each other). An interval of approximately 1 min was allowed between the measurements to avoid short-term fatigue for respiratory muscles. The higher of two reproducible values was considered in the data analysis [42].

#### 2.5.2. Dyspnea Level

Dyspnea level was measured by the MRC scale, which has been used for many years for grading the effect of breathlessness on daily activities [43]. This scale measures perceived respiratory disability according to the World Health Organization (WHO) definition of disability, which is “any restriction or lack of ability to perform an activity in the manner or within the range considered normal for a human being”. The MRC dyspnea scale was considered a simple tool to administer, allowing patients to indicate the extent to which their breathlessness affected their mobility [44]. The MRC dyspnea scale comprised a questionnaire that consisted of five statements concerning perceived breathlessness, such as grade 1—“I only get breathless with strenuous exercise”, grade 2—“I get short of breath when hurrying on the level or up a slight hill”, grade 3—“I walk slower than people of the same age on the level because of breathlessness or have to stop for breath when walking at my own pace on the level”, grade 4—“I stop for breath after walking 100 yards or after a few minutes on the level”, grade 5—“ I am too breathless to leave the house”. Patients selected the grade that applied to them [44].

#### 2.5.3. Health-Related Quality of Life

The impact of the disease on the patients’ health-related quality of life was assessed by the CAT, which is a self-administered questionnaire consisting of eight items assessing various disease manifestations showing adequate internal consistency (0.85–0.98) and test–retest reliability (0.80–0.96) [45].

### 2.6. Respiratory Muscle Training Intervention

The respiratory muscle training (RMT) was carried out using an Orygen Dual Valve device (Forumed S.L, Barcelona, CAT, Spain). This respiratory device allows patients to train the inspiratory and expiratory muscles simultaneously and to adjust the loads independently [46].

COVID-19 patients carried out RMT for 12 weeks with a frequency of 2 times per day in morning and afternoon and 5 days per week. All patients performed 5 series of 10 repetitions in each session with a rest of 1 min between-series. The resistance was provided by the Orygen Dual Valve, which allows individuals to exercise the inspiratory and expiratory muscles simultaneously during the training session [47]. Training was individually tailored for each participant.

The intensity of training was adjusted to 30% of PI_max_ and 30% of PE_máx_ during the first 6 weeks of training and to 40% of PI_max_ and 40% of PE_max_ during the last 6 weeks. All patients carried out domiciliary and daily self-training and visited the rehabilitation service weekly to re-adjust the training intensity by evaluating the PI_max_ and to report the presence of adverse effects such as increased fatigue, breathing problems, dizziness or sickness.

### 2.7. Sample Size Calculation

A sample size calculation was carried out within the G*Power 3.1.9.2 using *t* test family calculations by statistical tests for the means difference between 2 independent groups by the power analysis type of a priori sample size based on a given α of 0.05, power of 0.80 and large effect size of *d* > 0.80 [48]. According to these data, a total sample size of 40 participants, 20 participants for each group, was necessary.

### 2.8. Statistical Analysis

The 24.0 version of the Statistical Package for Social Sciences (SPSS) software (IBM; Armonk, NY, USA; IBM–Corp) was used to perform all statistical analyses using an α error of 0.05 and a statistically significant *p*-value < 0.05 according to a 95% confidence interval (CI).

For quantitative data, Shapiro–Wilk tests completed with the visual distribution of QQ graphs and histograms were used to assess the assumption of normality [49]. After, parametric data were detailed if *p*-values were ≥0.05 following Shapiro–Wilk tests. Non-parametric data were detailed if *p*-values were <0.05 following Shapiro–Wilk tests. Both data values were expressed as means ± standard deviations (SD) and 95% CIs, including lower and upper limits; mean differences and 95% CIs, also including lower and upper limits; *t* statistics reported for parametric analyses and *U* statistics reported for non-parametric data. Indeed, 95% CIs were preferred instead of values ranges because they provide a population value range consistent with a 95% level of confidence, thus, being appropriate for both parametric and non-parametric analyses and recommended if inferential statistical analyses are performed [50].

For intra-group comparisons, *p*-values from parametric data were obtained by the Student’s *t*-tests for paired samples. In addition, *p*-values from non-parametric data were obtained by the Wilcoxon tests for paired samples.

For inter-group comparisons, *p*-values from parametric data were obtained by the Student’s *t*-tests for independent samples according to the Levene’s tests for equality of variances. In addition, *p*-values from non-parametric data were obtained by the Mann–Whitney *U* tests for independent samples.

For all outcome measurement differences after RMT intervention, effect size was calculated by Cohen’s *d* and categorized in effect sizes as very small (*d* < 0.20), small (*d* = 0.20–0.49), medium (*d* = 0.50–0.79) or large (*d* > 0.8) [51].

In order to avoid the chance of a type I error, a 2-way analysis of variance (ANOVA) was carried out for repeated measurements over time (before and after intervention) as the within-subject factor and group (IMV and non-IMV) and as the between-group factor to compare main outcome measurements [52]. In addition, the Greenhouse–Geisser correction significance was used if the Mauchly tests rejected the sphericity [53]. Post-hoc comparisons were performed by Bonferroni’s corrections. The F-tests effect sizes were determined by the Eta squared (η^2^) coefficients and considered a small effect size if η^2^ = 0.01, medium effect size if η^2^ = 0.06, as well as large effect size if η^2^ = 0.14 [54].

Categorical data were expressed as percentages (%), frequencies (n), statistics (χ^2^) and *p*-values for between-groups comparisons by the Fisher exact tests for analyses of dichotomous data.

Finally, multivariate regression analyses were performed to predict the PI_max_ (%) as the main outcome measurement according to prior studies and recommendations [55,56]. Linear regression analyses were carried out using the stepwise selection method and *R*^2^ coefficients to establish the adjustment quality. Descriptive data and the other outcome measurements at baseline were selected as independent variables. PI_max_ (%) was selected as the dependent variable.

## 3. Results

### 3.1. Baseline Data

Descriptive data and outcome measurements at baseline were presented for the total sample (n = 40), as well as non-IMV (n = 20) and IMV (n = 20) groups in Table 1 and Table 2. There were between-groups statistically significant differences (*p* < 0.01) at baseline for fewer hospitalization days (mean difference = −34.65; 95% CI = −49.86–−19.45; *U* = 371.000) and lower MRC dyspnea scores (mean difference = −0.98; 95% CI = −1.59–−0.37; *U* = 314.000), as well as higher DLCO % (mean difference = 19.86; 95% CI = 9.16–30.56; *t* = 3.759) and PI_max_, measured in cmH_2_o (mean difference = 22.59; 95% CI = 6.83–38.35; *t* = 2.921) and % (mean difference = 29.36; 95% CI = 16.99–41.73; *t* = 4.805) in the non-IMV group with respect to the IMV group. The rest of the descriptive data and outcome measurements did not show statistically significant differences (*p* > 0.05) between both groups at baseline.

### 3.2. RMT Effect on the Total Sample of COVID-19 Patients

RMT effects on the outcome measurements of the total sample of COVID-19 patients are presented in Table 3 and show statistically significant differences (*p* < 0.05) with an effect size from small to large (*d* = 0.38–0.98) for higher PE_max_, measured in % (mean difference = 7.09; 95% CI = 1.13–13.04; *t* = 2.408), and PI_max_, measured in cmH_2_O (mean difference = 12.49; 95% CI = 2.70–22.27; *t* = 2.583) and % (mean difference = 15.51; 95% CI = 7.43–23.59; *W* = 143.000), as well as lower CAT score (mean difference = −5.34; 95% CI = −7.51–−3.17; *W* = 618.500) and MRC dyspnea score (mean difference = −0.83; 95% CI = −1.11– −0.56; *W* = 558.000) after 12 weeks of RMT with respect to baseline measurements. Nevertheless, PE_max_, measured in cmH_2_O, did not show statistically significant differences (*p* > 0.05) after RMT.

### 3.3. RMT Effect on the Non-IMV Group

RMT effects on the outcome measurements of the non-IMV group of COVID-19 patients are presented in Table 4 and showed statistically significant differences (*p* = 0.001) with a large effect size (*d* = 0.94–1.09) for lower CAT score (mean difference = −6.94; 95% CI = −9.90–−3.98; *W* = 165.000) and MRC dyspnea score (mean difference = −0.53; 95% CI = −0.80–−0.26; *W* = 118.000) after 12 weeks of RMT with respect to baseline measurements. Nevertheless, both PE_max_ and PI_max_ outcomes, measured in cmH_2_O and %, did not show any statistically significant differences (*p* > 0.05) after RMT.

### 3.4. RMT Effect on the IMV Group 

RMT effects on the outcome measurements of the IMV group of COVID-19 patients are presented in Table 5 and show statistically significant differences (*p* < 0.05) with an effect size from moderate to large (*d* = 0.54–1.13) for greater PI_max_, measured in cm H_2_O (mean difference = 19.01; 95% CI = 6.54–31.48; *t* = 3.192) and % (mean difference = 17.47; 95% CI = 7.88–27.10; *t* = 3.811), as well as lower CAT score (mean difference = −3.74; 95% CI = −7.01–0.47; *W* = 149.000) and MRC dyspnea score (mean difference = −1.13; 95% CI = −1.60–−0.66; *W* = 0.000) after 12 weeks of RMT with respect to baseline measurements. Nevertheless, PE_max_, measured in cmH_2_O and %, did not show statistically significant differences (*p* > 0.05) after RMT.

### 3.5. RMT Effect Comparison between Non-IMV and IMV Groups

The comparison of the outcome measurement differences obtained after 12 weeks of RMT between the non-IMV and IMV groups of COVID-19 patients is presented in Table 6 and shows statistically significant differences (*p* = 0.020) with a medium effect size (*d* = 0.74) for a greater MRC dyspnea score reduction (mean difference = 0.599; U = 114.000; 95% CI = 0.77–1.12) in the IMV group with respect to the non-IMV group. Nevertheless, the rest of the outcome measurements do not show any statistically significant differences (*p* > 0.05) between both non-IMV and IMV groups.

**Figure 1:**
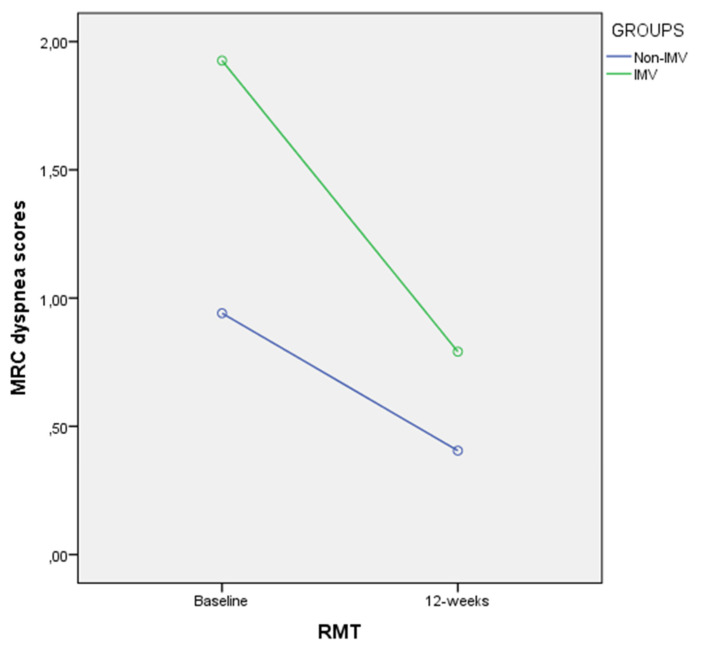
Linear graph showing the comparison of outcome measurement differences after RMT between non-IMV and IMV groups. Abbreviations: IMV, invasive mechanical ventilation; RMT, respiratory muscle training; MRC, Medical Research Council dyspnea scale.

These findings were confirmed by the 2-way ANOVA for repeated measurements. First, PE_max_ in cm H_2_O did not show statistically significant differences for time (*p* = 0.127; F = 2.437; η^2^ = 0.060) nor time×group interaction (*p* = 0.324; F = 0.997; η^2^ = 0.026). Furthermore, ANOVA for repeated measurements for PE_max_ in % displayed significant differences for time (*p* = 0.022; F = 5.670; η^2^ = 0.130) but not for time×group interaction (*p* = 0.702; F = 0.149; η^2^ = 0.004). Second, ANOVA for repeated measurements for PI_max_ in cm H_2_O showed significant differences for time (*p* = 0.013; F = 6.820; η^2^ = 0.152) but not for time×group interaction (*p* = 0.180; F = 1.862; η^2^ = 0.047). Furthermore, PI_max_ in % presented significant differences for time (*p* < 0.001; F = 14.798; η^2^ = 0.280) but not for time×group interaction (*p* = 0.627; F = 0.240; η^2^ = 0.006). Third, CAT scores displayed significant differences for time (*p* < 0.001; F = 25.685; η^2^ = 0.403) but not for time×group interaction (*p* = 0.138; F = 3.301; η^2^ = 0.057). Finally, ANOVA for repeated measurements for MRC scores showed statistically significant differences for time (*p* < 0.001; F = 42.082; η^2^ = 0.525) as well as for time×group interaction (*p* = 0.025; F = 5.407; η^2^ = 0.125). Indeed, the Bonferroni‘s comparison confirmed the MRC differences obtained after 12 weeks of RMT between the non-IMV and IMV groups of COVID-19 patients, showing that the MRC dyspnea scores were reduced in the IMV group versus the non-IMV group (Figure 1).

### 3.6. Multivariate Regression Analysis for PI_max_ (%) Prediction

A linear regression analysis was performed in order to predict the PI_max_ (%) as the primary outcome measurement. Indeed, the PI_max_ (%) reduction was only predicted by the IMV presence (*R*^2^ = 0.378; β = −29.361; F _[1,38]_ = 23.089; *p* < 0.001). Thus, the rest of independent variables were excluded (*p* > 0.05) from this prediction model due to PI_max_ (%), as the dependent variable was not influenced or predicted by non-descriptive data or the other outcome measurements according to the pre-established F probability (*P*_in_ = 0.05, *P*_out_ = 0.10).

## 4. Discussion

To the authors’ knowledge, this is the first case-series study comparing RMT effects in COVID-19 patients who received IMV compared with patients who did not receive IMV. Indeed, specific low intensity RMT may provide beneficial effects to improve respiratory muscle strength, health-related quality of life and dyspnea in patients who suffered from COVID-19. Specifically, the application of low intensity RMT in COVID-19 patients who were under IMV in an ICU could decrease dyspnea levels, in conjunction with a tendency to increase PI_max_ with respect to COVID-19 patients who did not receive IMV nor stay in an ICU.

In accordance with our research, a recent study carried out by Abodonya et al. [57] applied RMT for 2 weeks, generating an improvement in COVID-19 patients who received IMV in an ICU regarding pulmonary functional parameters, dyspnea scores, functional performance tests and quality of life. These authors concluded that an RMT program should be recommended for COVID-19 patients who stayed in an UCI. Nevertheless, these authors did not compare their findings with respect to COVID-19 patients who did not receive IMV nor an ICU stay. In addition, our case-series study used an incremental RMT protocol with an intensity of training adjusted from 30% of PI_max_ and 30% of PE_max_ for 6 weeks to 40% of PI_max_ and 40% of PE_max_ for 6 weeks [46], while Abodonya et al. [57] used a non-incremental protocol using a threshold load with 50% of PI_max_ for only 2 weeks.

At baseline, our study demonstrated that a higher dyspnea score and lower inspiratory muscle strength presented in COVID-19 patients who received IMV in an ICU with respect to COVID-19 patients who were not under IMV nor an ICU stay. In this line, IMV effects are well-known regarding the dyspnea level increase and the reduction in inspiratory muscle strength [58], and our study supported these findings in COVID-19 patients under IMV who stayed in an ICU.

After RMT intervention, the total sample of COVID-19 patients improved all outcome measurements. Indeed, patients who received IMV improved inspiratory muscle strength, quality of life and dyspnea, while patients who did not receive IMV did not improve inspiratory muscle strength. A dyspnea reduction presented after RMT between COVID-19 patients who received IMV versus patients who were not under IMV, and in addition, a reduction in inspiratory muscle strength was only predicted by the presence of IMV during an ICU stay. Despite the lack of similar research studies, the research carried out by Abodonya et al. [57] supported our study findings concerning RMT in COVID-19 patients who underwent IMV in an ICU, as described previously.

### 4.1. Future Studies

According to RMT intervention on other conditions, such as heart failure [59,60] or common pathologies suffered in an ICU [33], future clinical trials should compare low versus high intensity RMT on respiratory muscle strength in these patients who suffered from IMV in an ICU. In addition, a novel study suggested that RMT was feasible and could reduce respiratory complications in neurological patients with COVID-19, claiming the necessity of further studies in this research line [61].

### 4.2. Limitations

This study followed a case-series design due to the long duration of 12 weeks of RMT in COVID-19 patients with and without IMV in the recent pandemic. Authors recognize that this is a main limitation, and randomized clinical trials should be performed in the near future in order to determine RMT effectiveness. According to the lack of a specific tool to measure quality of life in COVID-19 patients during the recruitment date, the CAT was used as the most suitable tool to measure health related quality of life in our study, but we acknowledge its limitations in these patients because this questionnaire was initially designed to assess COPD patients [45]. Finally, although multivariate linear regression analyses were carried out to predict the PI_max_ (%) as the main outcome measurement following prior studies and using the *R*^2^ or “goodness of fit” to assess how good the fitted model was at explaining the observed data [55,56], future studies should analyze another aim to select the model that best predicts the outcome using new data by Bayesian models information criteria, such as AIC (applicable information criterion) or WAIC (widely applicable information criterion) [62].

## 5. Conclusions

Low intensity RMT may improve respiratory muscle strength, health related quality of life and dyspnea in COVID-19 patients. Specifically, low intensity RMT could improve dyspnea level and maybe PI_max_ in COVID-19 patients who received IMV in an ICU. Future clinical trials should compare low versus high intensity RMT on respiratory muscle strength in these patients who suffered from IMV in an ICU.

## Figures and Tables

**Table 1:** Quantitative data for COVID-19 patients with and without IMV at baseline.

Quantitative Data at Baseline	Total Sample (n = 40) Mean ± SD (95% CI)	Non-IMV (n = 20) Mean ± SD (95% CI)	IMV (n = 20) Mean ± SD (95% CI)	Mean Difference (95% CI)	Statistics	*p*-Value
Age (years)	56.0 ± 11.3 (52.3–59.6)	56.4 ± 12.0 (50.7–62.0)	55.6 ± 10.8 (50.5–60.7)	0.7 (−6.6–8.1)	*t* = 0.206	0.838 *
Hospitalization (days)	30.6 ± 28.8 (21.4–39.8)	13.3 ± 9.1 (9.0–17.5)	47.9 ± 31.4 (33.2–62.6)	−34.6 (−49.8–−19.4)	*U* = 371.000	**<0.001 ^†^**
ICU (days)	15.7 ± 23.5 (8.2–23.2)	N/A	31.5 ± 24.7 (19.9–43.1)	N/A	N/A	N/A
FVC (%)	78.8 ± 15.3 (73.9–83.8)	80.9 ± 14.0 (74.3–87.4)	76.8 ± 16.6 (69.0–84.6)	4.0 (−5.8–13.9)	*U* = 169.500	0.414 ^†^
FEV1(%)	88.5 ± 16.5 (83.2–93.8)	88.4 ± 14.4 (81.6–95.1)	88.7 ± 18.9 (79.9–97.6)	−0.3 (−11.1–10.3)	*U* = 205.500	0.883 ^†^
IT (%)	86.5 ± 6.3 (84.5–88.6)	84.9 ± 7.0 (81.5–88.2)	88.2 ± 5.1 (85.8–90.6)	−3.3 (−7.3–0.3)	*t* = −1.735	0.091 *
DLCO (%)	75.1 ± 19.3 (68.9–81.2)	85.0 ± 19.5 (75.9–94.1)	65.1 ± 13.3 (58.9–71.4)	19.8 (9.1–30.5)	*t* = 3.759	0.001 *
DL/VA (%)	89.1 ± 15.4 (84.1–94.0)	91.7 ± 18.7 (82.8–100.3)	86.6 ± 11.1 (81.4–91.8)	4.9 (−4.9–14.9)	*t* = 1.018	0.317 *
PE_max_ (cm H2O)	99.5 ± 38.0 (87.3–111.7)	101.1 ± 37.1 (83.7–118.5)	97.9 ± 39.8 (79.2–116.6)	3.2 (−21.4–27.8)	*t* = 0.263	0.794 *
PE_max_ (%)	50.1 ± 16.3 (44.9–55.4)	52.9 ± 16.1 (45.3–60.4)	47.4 ± 16.6 (39.6–55.2)	5.4 (−5.0–15.9)	*t* = 1.048	0.301 *
PI_max_ (cm H_2_O)	71.3 ± 26.7 (62.8–79.8)	82.6 ± 29.3 (68.9–96.3)	60.0 ± 18.3 (51.4–68.6)	22.5 (6.8–38.3)	*t* = 2.921	0.006 *
PI_max_ (%)	70.4 ± 24.1 (62.7–78.2)	85.1 ± 22.0 (74.8–95.4)	55.7 ± 16.0 (48.2–63.3)	29.3 (16.9–41.7)	*t* = 4.805	<0.001 *
CAT (scores)	12.1 ± 8.2 (9.4–14.7)	10.3 ± 7.0 (7.0–13.6)	13.8 ± 9.0 (9.6–18.0)	−3.5 (−8.7–1.7)	*U* = 254.000	0.149 ^†^
MRC (scores)	1.4 ± 1.0 (1.0–1.7)	0.9 ± 0.7 (0.6–1.2)	1.9 ± 1.1 (1.3–1.9)	−0.9 (−1.5–−0.3)	*U* = 314.000	**0.002 ^†^**

Abbreviations: CAT, COPD assessment test; CI, confidence interval; DLCO, diffusing capacity for carbon monoxide; DL/VA, diffusing capacity divided by the alveolar volume; FVC, forced vital capacity; FEV1, forced expiration volume in the 1st second; ICU, intensive care unit; IMV, invasive mechanical ventilation; IT, Tiffeneau index; MRC, Medical Research Council dyspnea scale; N/A, not applicable; PE_max_, maximal expiratory pressure; PI_max_, maximal inspiratory pressure; SD, standard deviation. * Student’s *t*-test for independent samples used. ^†^ Mann–Whitney *U* test applied. For all analyses, *p* < 0.05 (for a confidence interval of 95%) was considered statistically significant (**in bold**).

**Table 2:** Categorical data for COVID-19 patients with and without IMV at baseline.

Categorical Data at Baseline		Total Sample (n = 40) n (%)	Non-IMV (n = 20) n (%)	IMV (n = 20) n (%)	Statistics	*p*-Value ^†^
Sex	Female	26 (65%)	12 (60%)	14 (70%)	χ^2^ = 0.440	0.741
	Male	14 (35%)	8 (40%)	6 (30%)		
Hypertension	No	24 (60%)	13 (65%)	11 (55%)	χ^2^ = 0.417	0.748
	Yes	16 (40%)	7 (35%)	9 (45%)		
Diabetes	No	33 (82.5%)	18 (90%)	15 (75%)	χ^2^ = 1.558	0.407
	Yes	7 (17.5%)	2 (10%)	5 (25%)		
Dyslipidemia	No	34 (85%)	19 (95%)	15 (75%)	χ^2^ = 3.137	0.182
	Yes	6 (15%)	1 (5%)	5 (25%)		
CAD	No	37 (92.5%)	17 (85%)	20 (100%)	χ^2^ = 3.243	0.231
	Yes	3 (7.5%)	3 (15%)	0 (0%)		
COPD	No	36 (90%)	17 (85%)	19 (95%)	χ^2^ = 1.111	0.605
	Yes	4 (10%)	3 (15%)	1 (5%)		
Smoker	No	31 (77.5%)	16 (80%)	15 (75%)	χ^2^ = 0.143	1.000
	Yes	9 (22.5%)	4 (20%)	5 (25%)		
Obesity	No	36 (90%)	19 (95%)	17 (85%)	χ^2^ = 1.111	0.605
	Yes	4 (10%)	1 (5%)	3 (15%)		
CKD	No	30 (75%)	16 (80%)	14 (70%)	χ^2^ = 0.533	0.716
	Yes	10 (25%)	4 (20%)	6 (30%)		
Hypothyroidism	No	39 (97.5%)	19 (95%)	20 (100%)	χ^2^ = 1.026	1.000
	Yes	1 (2.5%)	1 (5%)	0 (0%)		

Abbreviations: CAD, coronary artery disease; COPD, chronic obstructive pulmonary disease; CKD, chronic kidney disease; IMV, invasive mechanical ventilation; N/A, not applicable. ^†^ Fisher exact tests were used. For all analyses, *p* < 0.05 (for a confidence interval of 95%) was considered statistically significant.

**Table 3:** RMT effect on the outcome measurements of the total sample of COVID-19 patients.

Outcome Measurements	Baseline (n = 40) Mean ± SD (95% CI)	After RMT (n = 40) Mean ± SD (95% CI)	Mean Difference (95% CI)	Statistics	*p*-Value	Effect Size (Cohen *d*)
PE_max_ (cm H_2_O)	99.5 ± 38.0 (87.3–111.7)	108.9 ± 34.0 (98.0–119.8)	9.3 (−2.7–21.5)	*W* = 288.000	0.101 ^†^	*d* = 0.24
PE_max_ (%)	50.1 ± 16.3 (44.9–55.4)	57.2 ± 16.9 (51.8–62.6)	7.0 (1.1–13.0)	*t* = 2.408	0.021 *	*d* = 0.38
PI_max_ (cm H_2_O)	71.3 ± 26.7 (62.8–79.8)	83.8 ± 31.8 (73.6–94.0)	12.4 (2.7–22.2)	*t* = 2.583	0.014 *	*d* = 0.40
PI_max_ (%)	70.4 ± 24.1 (62.7–78.2)	85.9 ± 33.1 (75.3–96.5)	15.5 (7.4–23.5)	*W* = 143.000	**<0.001 ^†^**	*d* = 0.61
CAT (scores)	12.1 ± 8.2 (9.4–14.7)	6.7 ± 6.7 (4.6–8.9)	−5.3 (−7.5–−3.1)	*W* = 618.500	**<0.001 ^†^**	*d* = 0.78
MRC (scores)	1.4 ± 1.0 (1.0–1.7)	1.0 ± 4.2 (1.0–1.7)	−0.8 (−1.1–−0.5)	*W* = 558.000	**<0.001 ^†^**	*d* = 0.97

Abbreviations: CAT, COPD assessment test; CI, confidence interval; RMT, respiratory muscle training; MRC, Medical Research Council dyspnea scale; PE_max_, maximal expiratory pressure; PI_max_, maximal inspiratory pressure; SD, standard deviation. * Student’s *t*-test for paired samples was used. ^†^ Wilcoxon test for paired samples was applied. For all analyses, *p* < 0.05 (for a confidence interval of 95%) was considered statistically significant (**bold**).

**Table 4:** RMT effect on the non-IMV group outcome measurements.

Outcome Measurements (Scores)	Baseline (n = 20) Mean ± SD (95% CI)	After RMT (n = 20) Mean ± SD (95% CI)	Mean Difference (95% CI)	Statistics	*p*-Value	Effect Size (Cohen *d*)
PE_max_ (cm H_2_O)	101.1 ± 37.1 (83.7–118.5)	104.5 ± 29.5 (90.7–118.3)	3.3 (−12.7–19.4)	*W* = 99.000	0.823 ^†^	*d* = 0.09
PE_max_ (%)	52.9 ± 16.1 (45.3–60.4)	58.8 ± 17.3 (50.7–66.9)	5.9 (−1.1–13.0)	*W* = 69.000	0.179 ^†^	*d* = 0.39
PI_max_ (cm H_2_O)	82.6 ± 29.3 (68.9–96.3)	88.6 ± 32.5 (73.3–103.8)	5.9 (−9.7–21.6)	*t* = 0.797	0.435 *	*d* = 0.17
PI_max_ (%)	85.1 ± 22.0 (74.8–95.4)	98.6 ± 35.1 (82.2–115.1)	13.5 (−0.3–27.4)	*W* = 54.000	0.057 ^†^	*d* = 0.45
CAT (scores)	10.3 ± 7.0 (7.0–13.6)	3.4 ± 3.6 (1.6–5.1)	−6.9 (−9.9–−3.9)	*W* = 165.000	**0.001 ^†^**	*d* = 1.09
MRC (scores)	0.9 ± 0.7 (0.6–1.2)	0.4 ± 0.3 (0.2–0.5)	−0.5 (−0.8–−0.2)	*W* = 118.000	**0.001 ^†^**	*d* = 0.94

Abbreviations: CAT, COPD assessment test; CI, confidence interval; RMT, respiratory muscle training; IMV, invasive mechanical ventilation; MRC, Medical Research Council dyspnea scale; PE_max_, maximal expiratory pressure; PI_max_, maximal inspiratory pressure; SD, standard deviation. * Student’s *t*-test for paired samples was used. ^†^ Wilcoxon test for paired samples was applied. For all analyses, *p* < 0.05 (for a confidence interval of 95%) was considered statistically significant (**bold**).

**Table 5:** RMT effect on the IMV group outcome measurements.

Outcome Measurements	Baseline (n = 20) Mean ± SD (95% CI)	After RMT (n = 20) Mean ± SD (95% CI)	Mean Difference (95% CI)	Statistics	*p*-Value	Effect Size (Cohen *d*)
PE_max_ (cm H_2_O)	97.9 ± 39.8 (79.2–116.6)	113.3 ± 38.3 (95.4–131.2)	15.4 (−3.9–34.7)	*t* = 1.663	0.113 *	*d* = 0.37
PE_max_ (%)	47.4 ± 16.6 (39.6–55.2)	55.7 ± 16.8 (47.8–63.6)	8.2 (−1.9–18.4)	*t* = 1.685	0.108 *	*d* = 0.37
PI_max_ (cm H_2_O)	60.0 ± 18.3 (51.4–68.6)	79.0 ± 31.2 (64.4–93.7)	19.0 (6.5–31.4)	*t* = 3.192	0.005 *	*d* = 0.71
PI_max_ (%)	55.7 ± 16.0 (48.2–63.3)	73.2 ± 26.0 (61.0–85.4)	17.4 (7.8–27.1)	*t* = 3.811	0.001 *	*d* = 0.85
CAT (scores)	13.8 ± 9.0 (9.6–18.0)	4.4 ± 3.6 (1.6–5.1)	−3.7 (−7.0–0.4)	*W* = 149.000	**0.029 ^†^**	*d* = 0.54
MRC (scores)	1.9 ± 1.1 (1.3–1.9)	0.7 ± 4.2 (0.5–1.0)	−1.1 (−1.6–−0.6)	*W* = 0.000	**<0.001 ^†^**	*d* = 1.13

Abbreviations: CAT, COPD assessment test; CI, confidence interval; RMT, respiratory muscle training; IMV, invasive mechanical ventilation; MRC, Medical Research Council dyspnea scale; PE_max_, maximal expiratory pressure; PI_max_, maximal inspiratory pressure; SD, standard deviation. * Student’s *t*-test for paired samples was used. ^†^ Wilcoxon test for paired samples was applied. For all analyses, *p* < 0.05 (for a confidence interval of 95%) was considered as statistically significant (**bold**).

**Table 6:** Comparison of outcome measurement differences after RMT between non-IMV and IMV groups.

Outcome Differences after RMT	Non-IMV (n = 20) Mean ± SD (95% CI)	IMV (n = 20) Mean ± SD (95% CI)	Mean Difference (95% CI)	Statistics	*p*-Value	Effect Size (Cohen *d*)
PE_max_ (cm H_2_O)	3.3 ± 34.3 (−12.7–19.4)	15.4 ± 41.4 (−3.9–34.7)	−12.0 (−36.3–12.3)	*t* = −0.999	0.324 *	*d* = 0.31
PE_max_ (%)	5.9 ± 15.2 (−1.1–13.0)	8.2 ± 21.8 (−1.9–18.4)	−2.2 (−14.3–9.7)	*t* = −0.385	0.702 *	*d* = 0.12
PI_max_ (cm H_2_O)	5.9 ± 33.4 (−9.7–21.6)	19.0 ± 26.6 (6.5–31.4)	−13.0 (−32.4–6.3)	*t* = −1.365	0.180 *	*d* = 0.43
PI_max_ (%)	13.5 ± 29.6 (−0.3–27.4)	17.4 ± 20.5 (7.8–27.1)	−3.9 (−20.2–12.3)	*U* = 235.500	0.341 ^†^	*d* = 0.15
CAT (scores)	−6.9 ± 6.3 (−9.9–−3.9)	−3.7 ± 6.9 (−7.0–−0.4)	3.2 (−7.4–1.07)	*t* = −1.517	0.138 *	*d* = 0.48
MRC (scores)	−0.5 ± 0.5 (−0.8–−0.2)	−1.1 ± 1.0 (−1.6–−0.6)	0.5 (0.7–1.1)	*U* = 114.000	**0.020 ^†^**	*d* = 0.74

Abbreviations: CAT, COPD assessment test; CI, confidence interval; RMT, respiratory muscle training; IMV, invasive mechanical ventilation; MRC, Medical Research Council dyspnea scale; PE_max_, maximal expiratory pressure; PI_max_, maximal inspiratory pressure; SD, standard deviation. * Student’s *t*-test for independent samples used. ^†^ Mann–Whitney *U* test applied. For all analyses, *p* < 0.05 (for a confidence interval of 95%) was considered statistically significant (**bold**).

## Data Availability

Raw data are available upon corresponding author request.
